# Disability, mental health symptom recognition, and treatment-seeking in resident physicians with depressive symptoms

**DOI:** 10.1080/10872981.2026.2740874

**Published:** 2026-09-29

**Authors:** Karina Pereira-Lima, Katherine E. T. Ross, Elena Frank, Erene Stergiopoulos, Zoie C. Sheets, Srijan Sen, Lisa M. Meeks

**Affiliations:** a Department of Anesthesiology, University of Michigan Medical School, Ann Arbor, MI, USA; b Department of Psychology, University of Michigan, Ann Arbor, MI, USA; c Michigan Neuroscience Institute, University of Michigan Medical School, Ann Arbor, MI, USA; d Department of Psychiatry, University of Toronto, Toronto, Canada; e Departments of Internal Medicine and Pediatrics, University of Chicago, Chicago, IL, USA; f Eisenberg Family Depression Center, University of Michigan Medical School, Ann Arbor, MI, USA; g Department of Medical Education, College of Medicine, University of Illinois, Chicago, IL, USA; h Department of Family Medicine, University of Michigan Medical School, Ann Arbor, MI, USA

**Keywords:** Disability, depression, internship and residency, help-seeking behavior, physicians

## Abstract

**Introduction:**

Resident physicians are at high-risk for depressive symptoms, yet most of those meeting depression criteria do not seek care. Resident physicians with disabilities are at higher risk for depressive symptoms. However, their mental health treatment-seeking behaviours remain poorly understood. This study investigates associations between self-identified disability-status, mental health symptom recognition, and treatment-seeking among first-year residents with depressive symptoms.

**Methods:**

We analysed data from the 2020–2022 cohorts of the Intern Health Study, a longitudinal study of first-year resident physicians. Depressive symptoms were assessed quarterly with the Patient Health Questionnaire (PHQ-9); scores ≥10 indicated a positive depression screen. Residents who screened positive at least once during internship and identified their disability-status were included. Mental health symptom recognition and treatment-seeking were assessed via quarterly surveys throughout the first residency year. Associations between disability-status, symptom recognition, and treatment-seeking were examined using weighted logistic regression adjusting for sex and specialty-type. Post-stratification and attrition weights addressed nonrepresentative sampling and loss to follow-up. Sensitivity analyses excluded interns reporting psychological disabilities.

**Results:**

Of 5,062 interns, 1,386 (27.4%) screened positive for depression during internship; 940 identified their disability-status and were included (127 [13.5%] with disabilities). Compared to nondisabled interns, those with disabilities were significantly more likely to recognise mental health symptoms (adjusted weighted-OR = 4.5, 95% CI: 1.8–14.7) and seek treatment (adjusted weighted-OR = 3.2, 95% CI: 2.1–5.0). Results remained significant in sensitivity analyses excluding psychological disabilities.

**Conclusion:**

Among depressed first-year residents, self-identified disability-status was associated with significantly greater symptom recognition and treatment-seeking. These findings suggest disability-related experiences may be associated with reduced normalisation of depressive symptoms in residency, offering insights for reducing stigma and treatment barriers in medical training.

## Introduction

Medical training is characterised by high stress and risk for mental health problems, yet over half of resident physicians who meet the criteria for depression do not seek treatment [1]. Recognising the impact of depressive symptoms is a critical precursor to treatment-seeking and may become skewed in the context of residency training [2,3], where depressive symptoms could become normalised. Prior studies suggest that lack of programme access puts residents with disabilities at a higher risk for developing depressive symptoms [4]. However, the relationship between disability-status and mental health treatment-seeking behaviours remain poorly understood. This study investigates the associations between self-identified disability-status, mental health problem recognition, and treatment-seeking among first-year resident physicians with depressive symptoms.

## Methods

We analysed data from the 2020–2022 cohorts of the Intern Health Study [5], a longitudinal study of first-year resident physicians. Incoming residents from participating institutions completed a baseline survey 2 months before internship and quarterly surveys during internship. A total of 5,062 (37.2%) completed the baseline and were enroled.

Depressive symptoms were assessed at baseline and quarterly throughout the internship year with the Patient Health Questionnaire (PHQ-9) [6]. A score ≥ 10 was considered a positive screen for depression. Residents who screened positive for depression at least at one quarterly survey and who identified their disability-status at the final survey (‘yes’ or ‘no’ responses to the question ‘Are you a person with a disability [e.g., ADHD, learning, psychological, chronic health, mobility, hearing, etc]’) were included. Mental health symptom recognition and treatment-seeking were assessed with the question ‘If you had mental health problems since completing the last questionnaire (PAST 3 months), did you seek professional help for them (select all that apply)?’ Possible responses included ‘I have not had any mental health problems,’ ‘I have had some mental health problems, but I have not sought help,’ ‘I have consulted with my institution's employee assistance programme,’ ‘I have consulted with general practitioner,’ ‘I have consulted with a therapist (e.g., psychiatrist, psychologist, social worker);’ ‘I have been admitted to a psychiatric hospital.’ Mental health symptom recognition was defined as not endorsing the item ‘I have not had any mental health problems’ at any quarterly survey. Treatment-seeking was defined as endorsing any form of mental health treatment at least once during internship. Psychiatric hospitalisations were not classified as treatment-seeking since they are not necessarily voluntary; however, because all individuals reporting hospitalisation also reported other treatment types, they were included as treatment-seeking in analyses.

We assessed associations between disability-status and cumulative year-level indicators of symptom recognition and treatment-seeking using logistic regression accounting for factors previously associated with treatment-seeking among intern physicians (i.e., sex, specialty-type) [1], Sensitivity analyses excluding interns reporting psychological disabilities were performed.

In accordance with previously described methods [1,7], we implemented sample weights for all analyses to mitigate biases for two distinct selection mechanisms: nonrepresentative sampling (poststratification weights) and loss to follow-up (attrition weights) for all analyses. For post-stratification weights, we used national census data from the American Association of Medical Colleges (AAMC) on first-year residents as our target population. These high-coverage national data had response rates of 94.6%–95.1% during the study years and provided the national distribution of first-year residents by gender, race/ethnicity, specialty, and entering year (2020–2022) as our target population. We then used the R package *anesrake* [8] to generate post-stratification weights in three sequential steps: (I) cohort year; (II) specialty type (surgical vs. nonsurgical) within cohort year; (III) gender and race/ethnicity within specialty type and cohort year. Of 5062 residents enroled at baseline, 2631 (52%) completed the disability-status question and at least one quarterly depressive symptoms assessment. For attrition weights, we first conducted LASSO regression to identify which baseline characteristics significantly predicted the completion of the disability-status question and depressive symptoms assessment at follow-up surveys. Among those, gender, race/ethnicity, and specialty type significantly predicted follow-up completion. We then used the R package *twang* [9] to implement gradient-boosted models to estimate the propensity score of follow-up completion and extracted inverse-probability attrition weights. We calculated the final weights by multiplying post-stratification weights and attrition weights, derived from the full baseline sample regardless of depressive symptoms or disability-status, and Winsorized them at the 95th percentile to reduce the influence of extreme weights before scaling them so the weighted total would add to the true baseline sample size. We then used the *svdesign* function of the R package *survey* [10,11] to incorporate the generated survey weights to the present study sample.

All analyses were performed in R-version-4.2.2, with a 2-sided *P* < .05 deemed as statistically significant. This study was approved by the University of Michigan Institutional Review Board (HUM00033029) and all participants provided electronic written informed consent.

## Results

Of 5062 participating interns, 1386 (27.4%) met criteria for depression at least once during internship. Of these, 940 identified their disability-status (yes/no). After weighting, 127 (13.5%) were classified as having disabilities (Table 1).

**Table 1.** Characteristics of intern physicians screening positive for depression (PHQ-9 ≥ 10) by disability-status.

Characteristic	Residents with disabilities, unweighted (*N* = 121)	Residents without disabilities, unweighted (*N* = 819)	Residents with disabilities, weighted (*N* = 127)	Residents without disabilities, weighted (*N* = 813)
*Sex*				
Female	79 (65.3%)	524 (64.0%)	80 (62.8%)	481 (59.1%)
Male	40 (33.1%)	285 (34.8%)	47 (37.2%)	330 (40.6%)
Missing	2 (1.6%)	10 (1.2%)	0 (0.0%)	2 (0.2%)
Specialty-type*				
Surgical	17 (14.0%)	164 (20.0%)	14 (11.3%)	164 (20.2%)
Nonsurgical	104 (86.0%)	655 (80.0%)	113 (88.7%)	649 (79.8%)
*Disability type*				
Attention deficit/hyperactivity disorder	82 (67.8%)	NA	87 (68.5%)	NA
Chronic health disability	21 (17.4%)	NA	22 (17.3%)	NA
Deaf or hard of hearing	4 (3.3%)	NA	3 (1.6%)	NA
Learning disability	6 (5.0%)	NA	5 (3.9%)	NA
Mobility disability	2 (1.7%)	NA	2 (1.6%)	NA
Psychological disability	14 (11.6%)	NA	17 (13.4%)	NA
Visual disability	2 (1.7%)	NA	2 (1.6%)	NA
Other	5 (4.1%)	NA	5 (3.9%)	NA
Prefer not to disclose	1 (0.8%)	NA	1 (1.6%)	NA

^*^ Surgical specialties were assigned based on the American College of Surgeons classification and, in this sample of residents screening positive for depression, included Neurological Surgery, Obstetrics and Gynaecologist, Ophthalmology, Orthopaedic Surgery, Otolaryngology-Head and Neck Surgery, Surgery-General, and Urology. Nonsurgical specialties in this sample included Anaesthesiology, Child Neurology, Emergency Medicine, Family medicine, Internal Medicine, Internal Medicine/Emergency Medicine, Internal Medicine/Paediatrics, Internal Medicine/Psychiatry, Interventional Radiology-Integrated, Neurology, Pathology-Anatomical and Clinical, Paediatrics, Paediatrics/Medical Genetics, Paediatrics/Psychiatry/Child and Adolescent Psychiatry, Physical Medicine and Rehabilitation, Psychiatry, Psychiatry/Family Medicine, Radiology-Diagnostic, Transitional Year.

Compared to nondisabled interns, those with disabilities were more likely to recognise mental health symptoms (weighted-OR = 4.7, 95% CI: 2.0–15.6; sensitivity analysis: weighted-OR = 4.0, 95% CI: 1.6–13.3) and to seek treatment (weighted-OR = 3.3, 95% CI:2.2–5.0; sensitivity analysis: weighted-OR = 2.9, 95% CI: 1.9–4.5) (Figure 1). Importantly, after accounting for factors previously associated with treatment-seeking among training physicians, disabled interns remained significantly more likely to recognise symptoms (adjusted weighted-OR = 4.5, 95% CI: 1.8–14.7; sensitivity analysis: adjusted weighted-OR = 3.8, 95% CI: 1.5–12.4) and seek treatment (adjusted weighted-OR = 3.2, 95% CI: 2.1–5.0; sensitivity analysis: adjusted weighted-OR = 2.8, 95% CI: 1.8–4.4) than interns without disabilities.

**Figure 1.**
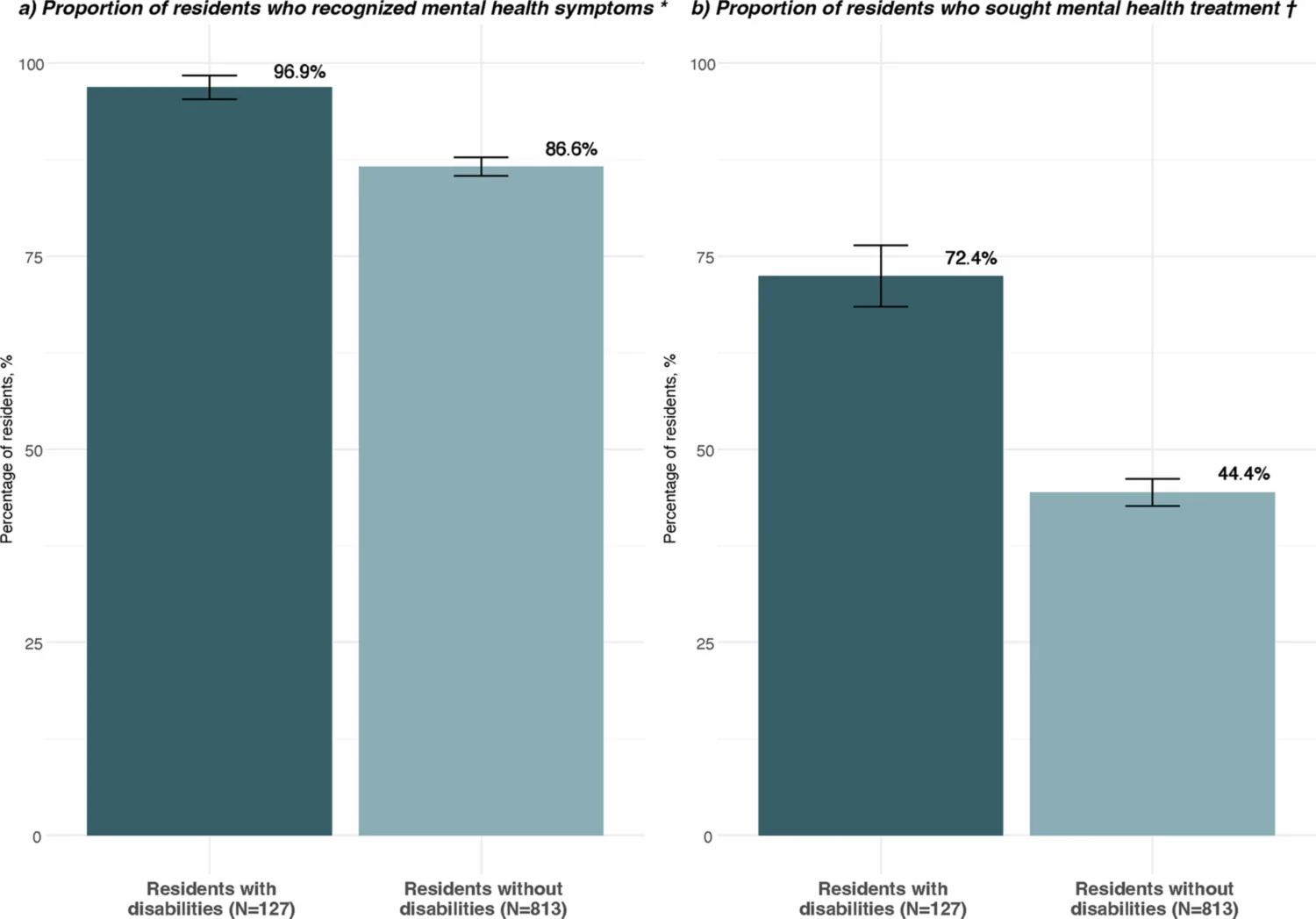
Mental health symptoms recognition and treatment seeking among interns screening positive for depression (PHQ-9 ≥ 10) by disability-status. *Statistically significant difference (unadjusted weighted-OR = 4.7, 95% CI: 1.9–15.6, *p* = .003). ^†^Statistically significant difference (unadjusted weighted-OR = 3.3, 95% CI: 2.2–5.0, *p* < .001).

## Discussion

Among first-year resident physicians screening positive for depression, those who self-identified with a disability were more likely to recognise mental health symptoms and seek mental health treatment than their non-disabled peers. These findings suggest that self-identified disability-status may be associated with reduced normalisation and greater recognition of depressive symptom impairment during residency [2,3], and encourage help-seeking behaviours once depressed. Familiarity with healthcare systems and self-advocacy [12] may contribute to this pattern, offering insights for reducing stigma and treatment barriers [13–16] in medical training. Given the associations of physician depressive symptoms with negative patient outcomes [17], future qualitative and larger prospective studies should explore potential drivers of increased symptom recognition and treatment-seeking among disabled trainees, implications for patient care, and heterogeneity across disability categories.

Limitations include self-reported measures, focus on first-year residents, possible unmeasured confounders, potential bias from item nonresponse, and inability to establish temporality or directionality. Further, although the prevalence of disabled interns and disability category distribution closely matched national AAMC data for the corresponding MD graduating classes [18], the modest number of disabled interns and predominance of ADHD led to imprecise estimates and precluded analyses by disability category. Nonetheless, this study contributes critical insights into the intersection of disability, mental health, and treatment-seeking in medical training and underscores the importance of leveraging lessons from disabled residents to create more supportive training environments.

## Data Availability

Deidentified data are publicly available via ICPSR: https://www.openicpsr.org/openicpsr/project/129225/.
